# Access to health care for persons with disabilities in rural South Africa

**DOI:** 10.1186/s12913-017-2674-5

**Published:** 2017-11-17

**Authors:** R. Vergunst, L. Swartz, K.-G. Hem, A. H. Eide, H. Mannan, M. MacLachlan, G. Mji, S. H. Braathen, M. Schneider

**Affiliations:** 1 Alan J Flisher Centre for Public Mental Health, Department of Psychology, Stellenbosch University, PO Box X1, Stellenbosch, Matieland 7602 South Africa; 2 SINTEF Technology and Society, Department of Health Research, PB 124 Blindern, 0314 Oslo, Norway; 3 Centre for Rehabilitation Studies, Stellenbosch University, Stellenbosch, South Africa; 4 School of Nursing, Midwifery & Health Systems, Health Sciences Centre, University College Dublin, Dublin, Ireland; 5 ALL Institute and Department of Psychology, Maynooth University, Maynooth, Ireland; 6 Olomouc University Social Health Institute, Palacký University Olomouc, Olomouc, Czech Republic; 7 Alan J Flisher Centre for Public Mental Health Department of Psychiatry & Mental Health, University of Cape Town, Cape Town, South Africa

**Keywords:** Disability, Rural, Health, Access, South Africa

## Abstract

**Background:**

Global research suggests that persons with disabilities face barriers when accessing health care services. Yet, information regarding the nature of these barriers, especially in low-income and middle-income countries is sparse. Rural contexts in these countries may present greater barriers than urban contexts, but little is known about access issues in such contexts. There is a paucity of research in South Africa looking at “triple vulnerability” – poverty, disability and rurality. This study explored issues of access to health care for persons with disabilities in an impoverished rural area in South Africa.

**Methods:**

The study includes a quantitative survey with interviews with 773 participants in 527 households. Comparisons in terms of access to health care between persons with disabilities and persons with no disabilities were explored. The approach to data analysis included quantitative data analysis using descriptive and inferential statistics. Frequency and cross tabulation, comparing and contrasting the frequency of different phenomena between persons with disabilities and persons with no disabilities, were used. Chi-square tests and Analysis of Variance tests were then incorporated into the analysis.

**Results:**

Persons with disabilities have a higher rate of unmet health needs as compared to non-disabled. In rural Madwaleni in South Africa, persons with disabilities faced significantly more barriers to accessing health care compared to persons without disabilities. Barriers increased with disability severity and was reduced with increasing level of education, living in a household without disabled members and with age.

**Conclusions:**

This study has shown that access to health care in a rural area in South Africa for persons with disabilities is more of an issue than for persons without disabilities in that they face more barriers. Implications are that we need to look beyond the medical issues of disability and address social and inclusion issues as well.

## Background

The United Nation’s *Convention on the Rights of Persons with Disabilities* (UNCRPD) is a human rights instrument on an international level intended to protect persons with disabilities’ dignity and rights. Eight guiding principles underlie the Convention, of which accessibility is one [1]. While access to health care is a major health issue [2], compromised access affects the performance of health care systems globally [3]. Health care needs that are not met and that exacerbate health disparities are experienced disproportionately by persons with disabilities [4]. Inequity in accessing health care for persons with disabilities is a global issue – in general, persons with disabilities have poorer health care access [5]. Political marginalisation, discrimination and inequitable access to health services are experienced by persons with disabilities resulting in poorer health outcomes [6].

According to Tomlinson et al. [7] and confirmed by the World Report on Disability [8], there is international evidence that persons with disabilities across the globe face distinctive barriers when accessing health care services, and show poorer health outcomes than nondisabled persons. Contemporary evidence continues to support the view that persons with disabilities have less access to health care [9–11]. Despite their frequent additional health care needs and limited access, persons with disabilities constitute a marginalised group in health services research. Their experiences within the health care system are not well understood, and research-based health service improvement interventions commonly exclude persons with disabilities [12].

This paper attempts to add to the health services research in exploring access to health care for persons with disabilities in a rural low-income context in South Africa.

### Disability and access to health care in low-income contexts

The majority of individuals with disability live in low-income contexts [8]. Recently, there has been an accumulation of evidence that barriers to health care access exist for persons with disabilities in less resourced countries. According to MacLachlan and Mannan [13], access to health care, even in wealthy countries, is often difficult for persons with disabilities, but in poorer countries the challenges are exacerbated, combining physical, financial, and attitudinal components. The EquitAble Project (see www.sintef.no/en/projects/equitable/) has documented a number of barriers to health care experienced by persons with disabilities in resource-poor settings in Africa [14–17].

### Rural access to health services

Concern over the availability of health services in rural areas has existed for decades [18] and “rural communities have long struggled to maintain access to quality health care services” [19] (p. 1). However, it is not enough, by itself, that a system of primary care be available in rural areas – the services must also be accessible [20]. One would expect that rural populations have reduced access to health care services compared to their urban counterparts, but according to Sibley and Weiner [21], studies have been contradictory and inconclusive.

Bourke, Humphreys, Wakerman, and Taylor [22] show that persons living in rural and remote areas face multiple challenges in accessing appropriate health services. These barriers to health care for the rural population have been well documented [23–27]. Rural communities share certain characteristics that affect both health and health care [28] and “do without ready access to the dense net of services – including health services – that characterises the urban environments” (p. 3). According to Rowland & Lyons (1989, cited in Schur & Franco) [29] (p. 25), some key characteristics for rural areas are:

1. poorly developed and fragile health infrastructures;
2. high prevalence rates for chronic illness and disability;
3. socioeconomic hardships; and
4. physical barriers such as distance and availability of transportation, including a lack of public transportation.

Access to quality health services becomes the single biggest issue, if not the defining issue, in rural health [30].

Disability and access to health care among the poor rural populations has received little attention. There is scarce data on their health needs [31]. What little literature there is suggests that persons with disabilities in rural areas have more problems and issues regarding their health care than persons with no disabilities in rural areas – especially when it comes to health care access [31].

### Disability in rural communities in South Africa

The right to access health care services in South Africa is guaranteed by Section 27 of the Constitution, but considerable inequities still remain, largely due to discrepancies in resource allocation [32, 33]. In their study on access to health care in South Africa, Harris et al. [34] concur with previous South Africa studies, confirming that poor, uninsured, Black Africans and rural groups have poorer access to health care than do other members of South African society [32, 33, 35, 36]. Only a few studies have looked at disability issues in rural South Africa [14, 37–40]. These studies focused specifically on disability and access to health care. More large scale quantitative research contributing to assessing and improving access to health care for persons with disabilities needs to be prioritised – especially in South African rural areas.

This study forms part of a larger Equitable study (www.sintef.no/en/projects/equitable/). The aim of this study was to compare persons with disabilities and persons with no disabilities in terms of access to health care in a rural impoverished area in South Africa. While international literature brings support to the existence of access barriers and inequitable health services, evidence is still limited when it comes to equal access for persons with disabilities. Based on the above literature on health disparities and access to health care, this article aims to reveal and compare specific access barriers in a rural context. The hypothesis is that persons with disabilities have poorer access to health care in a rural impoverished area in South Africa.

## Methods

### Context of study

Madwaleni is the third largest rural population in South Africa [41] with a 62% rural population. It is a deeply rural and impoverished area of the Eastern Cape Province, 220 km up the coast from East London, 100 km from Mthatha, 30 km from Elliotdale and 16 km from the Wild Coast. The Madwaleni area is situated in the rolling hills of the Amatole District within the Mbashe Municipality. This rural area is defined by poor infrastructure, lack of basic service provision, low levels of literacy, high levels of unemployment, limited access to health care and education, high incidence of communicable diseases and high mortality rates (Watermeyer & Barratt 2013, cited in Neille and Penn) [37].

### Sample of study

The sample in this observational cross-sectional study comprised of 773 individuals – 322 persons with disability and 451 controls (without disability) – covering 527 households. Children under the age of five were excluded from the sample. The age range was from 5 to 97. We used purposive sampling to first select four health centres surrounding the hospital, then random sampling to select the villages surrounding the health centres, and finally systematic sampling to select the households within the villages. Household questionnaires were administered with the head of the household to ascertain if the household had a person with a disability or not. Disability was defined by using the Washington Group Questions (WGQ) on Disability, wherein if an individual has “some difficulty” with two or more of the six questions, or has “a lot of difficulty” or is “unable to do” for one or more questions, they may be categorised as a person with activity or functional limitations, and categorised as “disabled”. Further details of the sampling and categorisation of participants have been reported by Eide et al. [42].

The Household questionnaire is a questionnaire administered to the head of the household in each household (after consent forms were completed). The Household questionnaire ascertained the composition of the household, i.e., the members of the household, and whether or not they had a disability using the Washington Group (WG) Questions on disability. If a person with a disability was identified in the household, then that person completed consent forms (relevant to age of persons; for example, Adult Consent Forms, Children Consent with parents and guardians forms and Assent Forms for children 15–17) and was also interviewed using the questionnaire for persons with disabilities. This questionnaire focused particularly on health and access to health care. If there were more than one person with a disability in a particular household, the person with the most severe disability was interviewed. This was ascertained by the disability rating scale in the household questionnaire where a higher total disability score according to the Washington Group method depicted more severe disability. This questionnaire focused particularly on health and access to health care. A third interview (after completion of consent forms) was carried out in the same household with a person without disability (in-house controls) matched to the person with disability by age (5 year latitude either way) and gender using a Control questionnaire. This questionnaire is a shortened version of the one administered to individuals with disability. If no matched non-disabled control was found in the household, no control interview was carried out in that household. If the household did not have a person with disability living in the house then this household became a neighbourhood control household. The head of the household would complete the Household questionnaire and a randomly selected person (using random tables) in the control household would complete the control questionnaire (neighbourhood controls). The sample used in the study was not a representative sample of the population. Characteristics of the Head of Household in this sample is that 66% of households were headed by women with an average age of 56 years. The average age for men-headed households was 55 years. Of the 532 households, there were 112 only case households, 175 only control households and 245 case and control households.

### Measures

Disability was defined by using the six questions developed by the Washington Group Questions on Disability [43]. The questions cover six domains: difficulty in hearing, seeing, walking, remembering, self-care and communicating. Answer alternatives are: i) no difficulty (0), ii) some difficulty (1), iii) a lot of difficulty (2), and iv) cannot do at all (3). For the purpose of this study, the six questions were added together to form a disability scale ranging from 0 (no difficulty in any of the six domains) to 18 (cannot do in all six domains). The scale had a mean value of 1.66 and standard deviation 2.27.

An asset scale was utilised to construct a measure on socio-economic status (SES). The respondents answered “yes” (1) or “no” (0) to the presence of 28 common items in the household, and the items were added together to form the asset scale. Range of the scale was then 0–28, mean value 3.52 and standard deviation 3.02.

### Instruments

Interviews using three questionnaires, depending on circumstances, were used in the study:

1. A Household questionnaire, comprising socio-demographic and socio-economic variables.
2. A Questionnaire for a person with a disability, with a range of disability relevant variables including questions on functional difficulties and access to services.
3. A Control questionnaire, which is a shortened version (in that there were no questions on assistive devices) of the questionnaire for persons with disability.

The survey questionnaires that were originally in English were translated into isiXhosa and back-translated to make them appropriate for the study site and its community members. The 17 data collectors/interviewers made use of cell phone technology and the translated questionnaires were programmed into the cell phone according to methods described by Tomlinson et al. [7]. The data capturing was recorded directly into the cell phone and these data were then sent to a central data base where it was collated and analysed. This method provided more accurate data, minimal missing data, was easier to monitor locally and remotely, and had built-in quality checks.

The approach to data analysis included quantitative data analysis using descriptive and inferential statistics. Frequency and cross tabulation, comparing and contrasting the frequency of different phenomena between persons with disabilities and persons with no disabilities, were used. Chi-square test, bi-variate and multivariate linear regressions were used to analyse the difference in barriers experienced by persons with and without disabilities.

## Results

Table 1 illustrates the sample characteristics of the study.

**Table 1:** Sample characteristics

Variables	*N*	%
Age:		
5–17	49	6.3
18–60	548	70.9
61 and over	176	22.8
Total	773	100.0
Gender:		
Male	209	27.0
Female	512	66.2
Missing data	52	6.8
Total	773	100.0
Education level (18+):		
No formal education	162	31.5%
Less than primary school	244	47.5%
Primary school	87	16.9%
Secondary school	18	3.5%
Tertiary level education	3	0.6%

The mean age for persons with disabilities was 54 years and persons without disabilities 33 years.

A higher percentage of persons with disabilities encountered barriers to accessing health care on a weekly and monthly basis. Of persons with no disabilities, 79.6% never had barriers to health care access compared to 70.8% of persons with disabilities (Table 2) (χ^2^ = 32.17, *p* < 0.001).

**Table 2:** How often has the availability of health care services and medical care been a problem for you? (*N* = 772)

		No disability	Disability
Barriers to Health Care	Never/not applicable	79.6% (312)	70.8% (267)
	Less than monthly	11.0 (43)	12.1 (46)
	Monthly	0.5% (2)	4.5% (17)
	Weekly	2.0% (8)	8.4% (17)
	Daily	6.9% (27)	4.2% (16)

Of persons with disabilities, 24.4% report that they did not get health services the last time they needed it, while the corresponding figure for non-disabled is 12.6% (χ^2^ = 17.77, *p* < .001).

Participants (*n* = 773) were given a list of 18 potential access barriers that they were asked to rate with response options of “No Problem” (score 1), “Small Problem” (score 2), “Moderate Problem” (score 3), “Serious Problem” (score 4) or “Insurmountable Problem” (score 5).

**Fig. 1:**
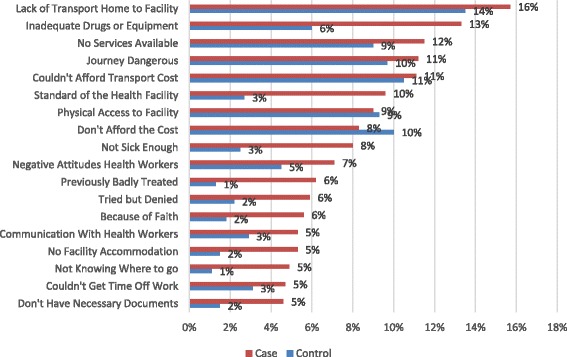
Access to Health Care-Madwaleni

In Fig. 1, the combined figures for “serious problems” and “insurmountable problems” are shown for both persons with and without disabilities. For all 18 items, more persons with than without disabilities report that the respective items are reasons for serious or insurmountable problems in accessing health care services.

The 18 items were subject to scale analyses (Alpha = 0.90). A principal component analyses gave support to a one-factor solution. The 18 items were then added together to form a “Barrier to health services” scale (range 18–62, mean value: 21.88, st.dev. 7.25). Mean value on the scale was 23.37 for persons with disabilities and 20.49 for non-disabled (F = 31.19, *p* < .001), indicating more experienced barriers among persons with disabilities.

Socio-economic status (SES) was measured by means of an Asset scale comprising 28 household items (1 = possess, 0 = do not possess) that were added together. In the Madwaleni sub-sample, minimum and maximum values on the scale ranged from 0 and 16, mean value 3.52 and standard deviation 3.02. Mean value among households with disabled persons was 3.70 and 3.33 among non-disabled households (F = 3.03, *p* = .08).

Mean number of household members was 4.4, and 4.6 and 4.3 in households with and without disabled members respectively (F = 3.21, *p* = .07).

A variable on household type (1 = have a disabled member, 2 = have no disabled member was included to control for household effect of disability.

The Washington Group 6 questions were used as an indicator of disability severity. All six items were added together, ranging from 6 to 24, mean value 7.7, standard deviation 2.27.

Time to get to health facility was registered by hours and minutes and ranged from 1 min to 20 h.

The bi-variate regressions (see Table 3) show that barriers to accessing health care services increase with age and severity of disability (score on WG 6), and reduce with increasing level of education and (near significant) with increasing number of members in the household. Further, being in a household without disabled members (household type) is associated with reduced barriers. No difference between males and females was revealed. The strongest association is the positive association between disability (WG 6) and barriers, closely followed by household type. Out of the six different items included in the Washington group questions used to determine disability, difficulties with seeing, hearing. Walking and remembering were all positively associated with increased barriers, while no association was found for difficulties with self-care and communication.

**Table 3:** Bivariate regressions on Barriers to health services (*N* = 762)

Variable	Beta	t	*p*
Age	0.08	2.13	<. 05
Sex	0.06	1.67	n.s.
Level of education	- 0.12	- 3.11	< .001
SES	- 0.42	- 1.15	n.s.
Disability (WG6 scale)	0.17	4.60	< .001
Type of household (1 = with disabled members, 2 = without disabled members)	- 0.16	- 4.32	< .001
Number of members in the household	- .07	- 1.82	.07
Difficulty (1–4)			
Seeing	0.13	3.62	< .001
Hearing	0.14	3.93	< .001
Walking	0.12	3.26	< .001
Remembering	0.13	3.62	< .001
Self-care	0.04	1.18	n.s.
Communicating	0.03	0.88	n.s.

In the multivariate model (see Table 4), the strongest predictors for reduced barriers to health services are type of household (with and without disabled members) and level of education. Belonging to a household without disabled member (s) and higher level of education reduce barriers. Increased disability severity and being female are associated with higher levels of barriers. Socio-economic status and number of household members remains non-significant . R^2^ for the entire model in 0.045.

**Table 4:** Multivariate regression of disability status, age, sex and level of education on barriers for accessing health services (*N* = 709)^a^

Variable	Beta	t	*P*
Age	- 0.08	- 1.79	.07
Sex	0.08	2.02	< .05n.s.
Level of education	- 0.09	- 2.24	< .05
Type of household (1 = with disabled members, 2 = without)	- .11	- 2.35	< .05
Disability () (WG6)	0.09	2.7	< .05
Total number in household	- .05	- 1.43	n.s.
SES	- .00	- .02	n.s.

^a^R ^2^ = 0.045

Comparing the specific difficulties, we found the highest figure for not getting health care the last time it was needed among persons with difficulties hearing (31.3%), followed by seeing difficulties (28.1%), walking difficulties (28.0%), difficulties with remembering (21.7%) and with self care (21.7%). For these difficulties significant differences were found between persons with and without the specific difficulties, and with the difference in percentage point ranging from 18 (hearing) to 4 (self-care).

## Discussion

Persons with disabilities in rural Madwaleni in South Africa faced significantly more barriers to accessing health care compared to persons without disabilities. The study’s hypothesis was thus confirmed by the findings. This is in accordance with, for instance, McDoom et al. [42] and Van Rooy et al. [16].

Transport-related issues were especially prominent – four of the top five barriers mentioned had to do with transport. In Madwaleni, transport is a particularly important issue because of the rugged terrain and great distances which need to be covered. Transport has been identified as a major issue in many other studies of this kind [31, 44–46].

The current study also showed that barriers to health care access increased with age, though this ceased to be significant in the multivariate analysis. Henning-Smith et al. [47] mention that older adults face barriers to care due to high health costs and lack of accessible transportation. This may be particularly true for older persons with disabilities who may face additional barriers to health care. Their study concluded that older adults with disabilities were more likely to experience barriers.

Education was shown to reduce barriers in Madwaleni. This may be because of the probable association between educational disadvantage and poverty.

Socio-economic status and total number of members in the households were not associated with barriers to health care. However, as the sample was drawn from a rather homogeneous and predominantly poor population, such household level characteristics vary less and will thus contribute less to explaining variation in the dependent variable.

Many persons with disabilities reported that they did not receive health care when they needed it and significantly fewer non-disabled reported the same. Persons with disabilities thus had higher rates of unmet health care needs. This is supported by the World Disability Report [8] as well as other sources [48, 49].

Increased understanding of the day-to-day challenges of persons with disabilities and their needs can educate those involved in health planning and care, especially “on how to incorporate various equities in order to create conditions that would enable the individuals with disabilities to achieve optimum health care” [50] (p. 258). It is clear from our study that the issue of access stretches far beyond questions about the health care system as narrowly understood – the questions of transport, poverty, and attitudinal barriers all need attention. In qualitative work as part of the same broad project, we have shown how attitudes towards disability may be intertwined with a lack of understanding of transport needs (see Vergunst et al.) [51].

There are limitations with this study. Being a quantitative study made it difficult to explore in depth the complexities and nuances of disability in terms of access to health care. The study is also descriptive and cross-sectional and does not develop or test interventions or causal pathways. Finally, the multivariate model, while demonstrating relevant associations, is limited in its explanation of barriers.

## Conclusion

This paper has highlighted that being a person with disability living in rural Madwaleni is not only about the “medical” issues, but more importantly about social and inclusion issues. As Swartz and Watermeyer [52] state, the story of disability in South Africa, as well as in other countries, is about social oppression. It is with this in mind that we need to shift and open our minds and ideas about disability (particularly with rural impoverished areas in South Africa), and that is a more complex situation. There is still much to do before persons with disabilities in general, and those living in rural impoverished areas in particular, can be included in all parts of society, including access to health care.

## Abbreviations

UNCRPD: United Nation’s *Convention on the Rights of Persons with Disabilities*
